# Co-ordinated Gene Expression in the Liver and Spleen during *Schistosoma japonicum* Infection Regulates Cell Migration

**DOI:** 10.1371/journal.pntd.0000686

**Published:** 2010-05-18

**Authors:** Melissa L. Burke, Donald P. McManus, Grant A. Ramm, Mary Duke, Yuesheng Li, Malcolm K. Jones, Geoffrey N. Gobert

**Affiliations:** 1 Molecular Parasitology Laboratory, Queensland Institute of Medical Research, Herston, Queensland, Australia; 2 The School of Population Health, The University of Queensland, Herston, Queensland, Australia; 3 Hepatic Fibrosis Laboratory, Queensland Institute of Medical Research, Herston, Queensland, Australia; 4 Parasite Cell Biology Laboratory, Queensland Institute of Medical Research, Herston, Queensland, Australia; 5 The School of Veterinary Science, The University of Queensland, St. Lucia, Queensland, Australia; McGill University, Canada

## Abstract

Determining the molecular events induced in the spleen during schistosome infection is an essential step in better understanding the immunopathogenesis of schistosomiasis and the mechanisms by which schistosomes modulate the host immune response. The present study defines the transcriptional and cellular events occurring in the murine spleen during the progression of *Schistosoma japonicum* infection. Additionally, we compared and contrasted these results with those we have previously reported for the liver. Microarray analysis combined with flow cytometry and histochemistry demonstrated that transcriptional changes occurring in the spleen were closely related to changes in cellular composition. Additionally, the presence of alternatively activated macrophages, as indicated by up-regulation of *Chi3l3* and *Chi3l4* and expansion of F4/80^+^ macrophages, together with enhanced expression of the immunoregulatory genes *ANXA1* and *CAMP* suggests the spleen may be an important site for the control of *S. japonicum*-induced immune responses. The most striking difference between the transcriptional profiles of the infected liver and spleen was the contrasting expression of chemokines and cell adhesion molecules. Lymphocyte chemokines, including the homeostatic chemokines *CXCL13*, *CCL19* and *CCL21*, were significantly down-regulated in the spleen but up-regulated in the liver. Eosinophil (*CCL11*, *CCL24*), neutrophil (*CXCL1*) and monocyte (*CXCL14*, *CCL12*) chemokines and the cell adhesion molecules *VCAM1*, *NCAM1*, *PECAM1* were up-regulated in the liver but unchanged in the spleen. Chemokines up-regulated in both organs were expressed at significantly higher levels in the liver. Co-ordinated expression of these genes probably contributes to the development of a chemotactic signalling gradient that promotes recruitment of effector cells to the liver, thereby facilitating the development of hepatic granulomas and fibrosis. Together these data provide, for the first time, a comprehensive overview of the molecular events occurring in the spleen during schistosomiasis and will substantially further our understanding of the local and systemic mechanisms driving the immunopathogenesis of this disease.

## Author Summary

Schistosomiasis is a significant cause of illness and death in the developing world. Inflammation and scarring in the liver and enlargement of the spleen (splenomegaly) are common features of the disease. Changes occurring in the spleen have the potential to influence the way in which the body deals with infection but the mechanisms driving these changes are not well characterised. In the present study we determined, for the first time, the gene expression profile of the mouse spleen during infection with *Schistosoma japonicum* and compared these results to those previously reported for the liver to determine if processes occurring in these organs co-operate to promote hepatic inflammation and granuloma formation. Our data indicated that gene expression in the spleen is related to the types of cells present and suggest that the spleen might be important in controlling schistosome-induced inflammation. Comparison of the liver and spleen showed that expression of cell signalling molecules (chemokines) was much higher in the liver, potentially promoting the recruitment of specific cell types to this organ, causing inflammation and scarring. The results from this study enhance our knowledge of the mechanisms that drive schistosome-induced splenomegaly and liver inflammation.

## Introduction

Schistosomiasis, characterised by extensive hepatic fibrosis and splenomegaly, is a significant cause of parasitic morbidity and mortality [1]. Although extensive studies have been carried out to identify the processes driving hepatic granulofibrotic response, the immunopathogenesis of schistosome-induced splenomegaly has been largely neglected.

Splenomegaly is a common feature of many infectious diseases and can lead to alterations in the splenic architecture as well as the inherent immunological function of the organ. Changes in the splenic architecture following *Leishmania* and some viral infections have been shown to influence the nature of the immune response to subsequent infections [2], [3]. Schistosome infections induce significant splenomegaly characterised by loss of definition between the red and white pulp [4], [5], [6], [7]. Additionally, schistosome infections are known to modify the nature of the immune response to a number of other pathologies, including allergic responses and other parasitic infections, by as yet undetermined mechanisms [8]. Furthermore, undefined processes occurring in the spleen during active schistosome infections enhance the granulofibrotic response occurring in the liver [5]. The precise molecular mechanisms and transcriptional modulations corresponding to these cellular and immunological changes, however, have not been fully evaluated. Characterising the molecular processes occurring in the spleen during schistosomiasis is an important research priority if we are to fully comprehend the immunopathogenesis of this disease and the mechanisms by which schistosome infections modulate the immune response to other pathogens.

The study presented here describes the use of whole genome microarray analysis combined with flow cytometry and histology, to provide a comprehensive profile of the transcriptional and cellular response occurring in the murine spleen during *Schistosoma japonicum* infection. As well, we compare and contrast these results with those we have previously reported for the liver during the progression of *S. japonicum* egg-induced granuloma formation and hepatic fibrosis [9]. Our results reveal that there is co-ordinated expression of chemokines and cell adhesion molecules in the liver and spleen that may regulate the recruitment of effector cells to the liver during schistosome infection. Additionally, we demonstrate the up-regulation of several immunomodulatory elements in the spleen that may be involved in the control of the immune response to *S. japonicum* infection. The results of microarray analysis, flow cytometry and histology of the livers of the *S. japonicum* infected mice used in the present study are available [9] and the liver gene expression data are in the public domain (NCBI's Gene Expression Omnibus; Series Accession Number: GSE14367). Taken together these results provide insight into the integrated molecular mechanisms driving the development of schistosome induced pathology.

## Materials and Methods

### Ethics Statement

All work was conducted with the approval of the Queensland Institute of Medical Research Animal Ethics Committee.

### Mice and Parasites

Full details of the time-course experiments undertaken on *S. japonicum*-infected mice are described elsewhere [9]. Methods related to the analysis of the spleen are outlined below. Livers and spleens from the same animals were used for the purposes of parasitological, histological, microarray and flow cytometry analyses.

Four to six week old female C57BL/6 mice (Animal Resource Centre, Canningvale, Australia) were percutaneously infected with 20 *S. japonicum* cercariae (Chinese mainland strain, Anhui population). Mice were euthanized at 4 (n = 7), 6 (n = 7) and 7 (n = 8) weeks post-infection (p.i.) and spleen tissue collected. Three additional mice were used as uninfected controls. An identical experiment was performed for flow cytometry (n = 5 per group). Total adult worm pairs per mouse were recorded as a measure of parasite burden and eggs per gram of liver was calculated as a measure of hepatic egg burden as reported [9], [10].

### Histological Assessment

Formalin fixed, paraffin embedded spleen sections were stained by haematoxylin and eosin to assess splenic structure. Within spleen tissue, eosinophils were identified by Giemsa staining; neutrophils by Leder stain (naphthol AS-D chloroacetate) [11]; and macrophages by immunoperoxidase staining for the macrophage specific cell surface marker F4/80 (Primary antibody: rat anti-mouse F4/80, Abcam, Cambridge, USA; Secondary antibody: biotin-conjugated anti-rat immunoglobulin, Jackson ImmunoResearch Laboratories, Inc, West Grove, USA. Detection: Streptavidin-HRP, Jackson ImmunoResearch Laboratories, Inc, West Grove, USA). Slides were scanned using an Aperio slide scanner (Aperio Technologies, Vista, USA). The number of eosinophils and neutrophils in the spleen of each mouse was quantified by calculating the average number of positively stained cells in 20 high power fields (×400). Positive staining for F4/80 was measured using Aperio's Spectrum Plus Software positive pixel count algorithm (Version 8.2; Aperio Technologies, Vista, USA).

### Flow Cytometry

Leukocytes were isolated from whole spleens as described [12]. Briefly, spleens were digested in collagenase D (1mg/ml; Roche Diagnostics, Mannheim, Germany) and DNAse I (0.5mg/mL; Roche Diagnostics, Mannheim, Germany) for 30 mins at 37°C. The tissue was then passed through a 70µm cell strainer (BD Falcon, Bedford, USA) and washed with FACS buffer (1% bovine serum albumin (w/v), 0.1% sodium azide (v/v) in phosphate buffered saline). Red blood cells were lysed with Gey's lysis solution. The solution was then underlayed with FACS buffer and centrifuged at 1300rpm for 5 mins. The resulting cell pellet was resuspended in FACS buffer and the cells counted.

Cells were stained for specific cell markers by first incubating with anti-Fc-receptorIII antibodies (Monoclonal antibody producing hybridoma; Clone: 24.G2) to block non-specific binding and then with commercially available fluorochrome-conjugated antibodies for 30 mins on ice (APC-anti-CD4, FITC-anti-CD8b and PE-anti-CD19: BD Pharminogen; FITC-anti-CD3, Miltenyi Biotec, Germany). Cells were defined as CD4^+^ T-cells (CD3^+^/CD4^+^), CD8^+^ T-cells (CD3^+^/CD8^+^) and B-cells (CD19^+^). Data were acquired on a FACS Calibur Flow Cytometer (BD Bioscience) and analysed using FlowJo Software (Treestar Inc) and GraphPad Prism, version 5.0 (GraphPad Software, San Diego, USA).

### RNA Isolation and Purification

Total RNA was extracted from spleen tissue as described [13]. Briefly, spleen tissue was homogenised in Trizol (Invitrogen, Carlsbad, USA) using a Qiagen Tissuelyser (Qiagen Inc., Valencia, USA). A portion of the homogenate was then processed by phase extraction with Trizol and by column chromatography using an RNeasy Mini Kit (Qiagen Inc, Valencia USA). RNA quantity was measured using the Nanodrop-1000 (Nanodrop Technologies, Wilmington, USA) and quality was assessed using an Agilent Bioanalyzer (Agilent Technologies, Foster City, USA).

### Pooling of Spleen RNA for Microarray Analysis and Real-Time PCR

Each mouse group was normalised by log transformation for egg burden and outliers were excluded on the basis of 95% confidence intervals as described [9]. An equal amount of total RNA from the spleens of four mice with the highest quality total RNA were pooled for cRNA and cDNA synthesis.

### Microarray Analysis

#### cRNA synthesis and whole genome microarray analysis

cRNA was synthesised using the Illumina Total Prep RNA Amplification kit (Ambion Inc., Austin, USA). Microarray analysis was performed using Illumina Mouse 6 version 1.1 Whole Genome Expression Chips and scanned using an Illumina BeadStation according to the manufacturer's instructions (Illumina, San Diego, USA). Two technical replicates were performed for each cRNA sample. Splenic gene expression data are publicly available (NCBI's Gene Expression Omnibus; Series Accession Number: GSE19525).

#### Data analysis

Quality control of microarray data was performed by examining intensity histograms of hybridisation efficiency and noise in BeadStudio (version 3; Illumina, San Diego, USA). Expression values were entered into GeneSpring GX version 7.3 (Agilent Technologies/Silicon Genetics, Foster City, USA) and normalised to the 50^th^ percentile. Values less than 0.01 were set to 0.01. Data were then normalised to uninfected controls and filtered for significant signal on the basis of detection score, a BeadStudio generated measure of the signal intensity of the gene relative to negative controls (d>0.949, which equates to a confidence value of p≤0.05). For a gene to be accepted, at least 4 of 8 hybridisations had to pass these filtering criteria. Differentially expressed genes were identified by analysis of variance (ANOVA, p≤0.05 using Benjamini and Hochberg correction for multiple testing). Hierarchical clustering with the Pearson correlation measure of similarity was performed on ANOVA filtered data to identify common patterns of temporal gene expression. Keyword based searches for specific gene names were used to identify chemokines and other genes of interest.

#### Comparison of liver and spleen microarray profiles

Venn diagrams comparing genes that passed all filtering criteria and were ≥2 fold up- or down-regulated in the spleen and liver [9] were constructed to identify genes that were:

- commonly up- or down-regulated in the spleen and liver
- enhanced in the spleen (i.e. up-regulated in the spleen while down-regulated or unchanged in the liver)
- enhanced in the liver (up-regulated in the liver and down-regulated or unchanged in the spleen)
- specifically down-regulated in the spleen
- specifically down-regulated in the liver

#### Identification of over-represented biological functions and signalling pathways

DAVID functional annotation analysis (DAVID Bioinformatics Resources 2008, National Institute of Allergy and Infectious Diseases (NIAID), NIH [14], [15]) was used to identify biological processes, molecular functions and signalling pathways in the DAVID knowledge data base over-represented by genes in each of the identified hierarchical clusters and gene lists generated from comparison of the liver and spleen data. Ranking of functional annotations in DAVID is based Modified Fischer's exact test (EASE score, p≤0.05) [14]. To identify groups of common terms, functional annotation clustering was applied. The overall importance of functional annotation clusters is ranked on the basis of enrichment score, the geometric mean of the p-values of each annotation term in the group [14]. An arbitrary cut off of ±2 fold change in expression was applied allowing identification of changes in gene expression with likely biological significance.

### Real-Time PCR

cDNA was synthesised from pooled splenic total RNA using a Quantitect Reverse Transcription kit (Qiagen Inc., USA). cDNA concentration was measured using a Nanodrop-1000 (Nanodrop Technologies, Wilmington, USA.). Real-time PCR was used to validate a subset of the microarray data. Forward and reverse primers were sourced from the literature [16], [17], [18] or designed using Primer-Blast software (http://www.ncbi.nlm.nih.gov/tools/primer-blast) (Table S1). Hypoxanthine phosphoribosyltranferase (HPRT) was used as a housekeeping gene [18]. Real time PCR was performed using SYBR Green master mix (Applied Biosystems, Warrington, UK) on a Corbett Rotor Gene 6000 (Corbett Life Sciences, Concorde, Australia). Rotor-Gene 6000 Series software (version 1.7), Microsoft Office Excel 2003 and GraphPad Prism Version 5.00 for Windows (San Diego, California USA) were used in the analysis of results. Correlations between microarray and real-time PCR results were assessed using Spearman's Rho measure of correlation in GraphPad Prism Version 5.00 for Windows.

### Statistical Analysis

Changes in parasitological, histological, real time PCR and flow cytometry data were assessed by One Way ANOVA with post hoc Tukey testing (p≤0.05) using the GraphPad Prism Version 5.00 for Windows (San Diego, California USA). Statistical analysis of microarray data was performed using GeneSpring GX (version 7.3.1). Correlations between microarray and real-time PCR data were measured using Spearman's Rho correlation in GraphPad Prism Version 5.00 for windows as described [19].

## Results

### Parasitological and Histological Analyses

Details of the parasitological burden and kinetics of granuloma formation and fibrosis in the livers of the *S. japonicum* infected mice used in these experiments are reported elsewhere [9]. Briefly, mice were moderately infected with an average of 5 worm pairs. Schistosome eggs were first observed in the liver at 4 weeks post infection (p.i.) and hepatic egg burden increased significantly thereafter (1-Way ANOVA, p≤0.05) [9]. The kinetics of granuloma formation and fibrosis were consistent with previous reports for *S. japonicum* [20], [21].

**Figure 1:**
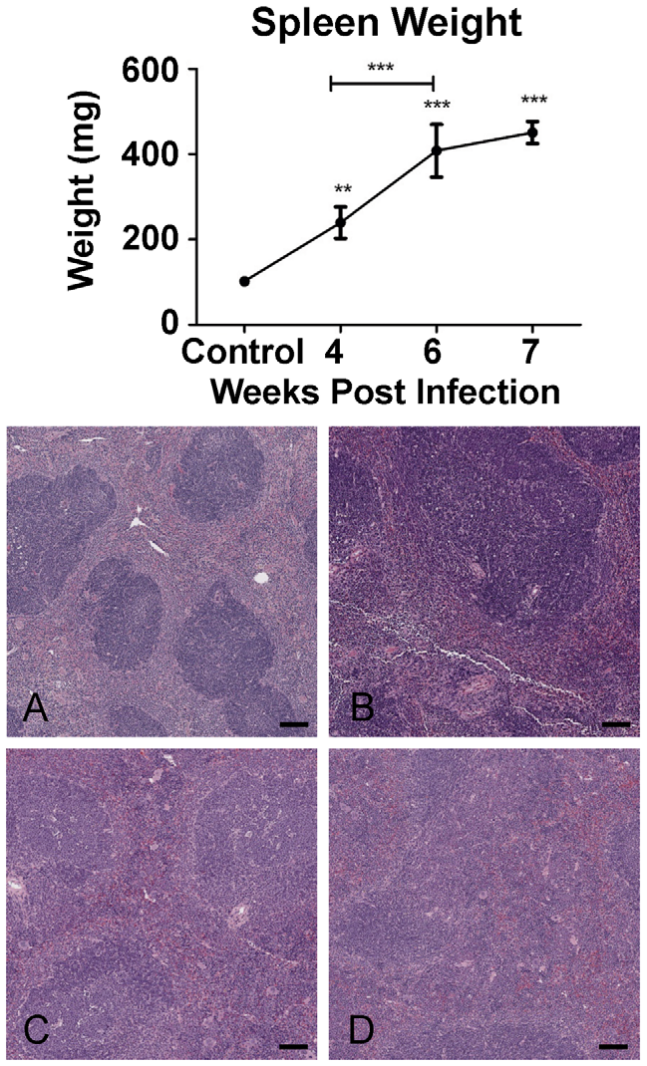
Splenic pathology induced by schistosome infection. Upper panel: Spleen weight increased over time following *S. japonicum* infection reaching approximately 4.5 times that of controls at 7 weeks p.i. The graph represents mean spleen weight of mice pooled for microarray analysis ±1SD (n = 4 per group). Lower panel: Schistosome induced splenomegaly was associated with increasing congestion of the red pulp and loss of definition between the red (lighter staining) and white pulp (darker staining) over time A: Control; B: 4 weeks p.i.; C: 6 weeks p.i.; D:7 weeks p.i. (Haematoxylin and eosin ×40; Bar equals 100µm.).

Total spleen weight increased significantly from 4 weeks p.i. onwards (Figure 1, upper panel). Changes to the splenic architecture were observed as early as 4 weeks p.i. and were characterised by increasing congestion of the red-pulp associated with loss of definition between the white and red pulp (Figure 1A–D, lower panel).

**Figure 2:**
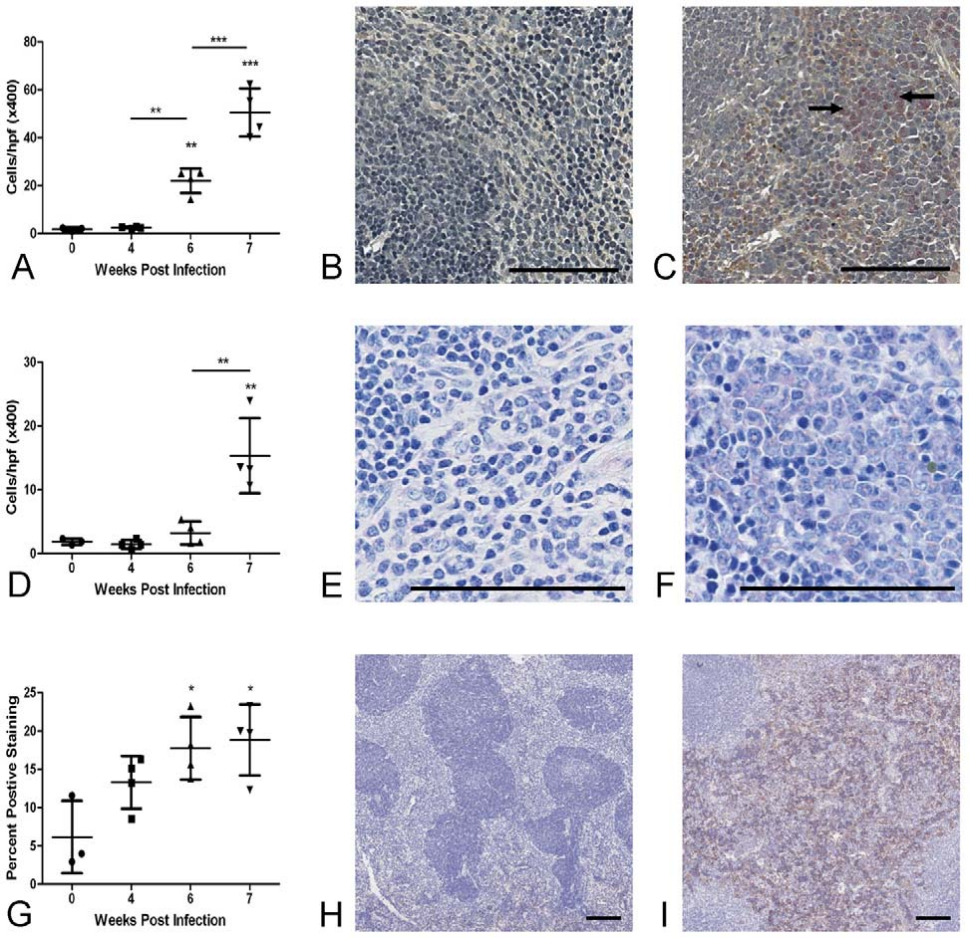
Splenomegaly is associated with accumulation of neutrophils, eosinophils and F4/80^+^ macrophages in the red pulp. There was a significant increases in the number of neutrophils (A–C: Leder stain for neutrophils (pink stain, arrowed) ×200 in uninfected control mice (B) and at 7 weeks p.i. (C)), eosinophils (D–F, Giemsa stain ×400 in uninfected control mice (E) and at 7 weeks p.i. (F)) and F4/80^+^ macrophages (G–I: F4/80 staining in uninfected control mice (H) and at 7 weeks p.i (I). (red-brown) ×40) in the splenic red pulp from as early as 6 weeks p.i. Values represent mean cells/high power field (eosinophils and neutrophils) or percent positive staining (F4/80) ±1SD of mice pooled for microarray analysis (n = 4 per group). Bar = 100µm.

The number of neutrophils in the splenic red pulp increased significantly from 6 weeks p.i. and eosinophil numbers were significantly elevated compared with uninfected mice at 7 weeks p.i. F4/80 staining for macrophages was significantly increased from 6 weeks p.i., reaching a peak of 19% total section area at 7 weeks p.i. (Figure 2A–I).

### Flow Cytometry

**Figure 3:**
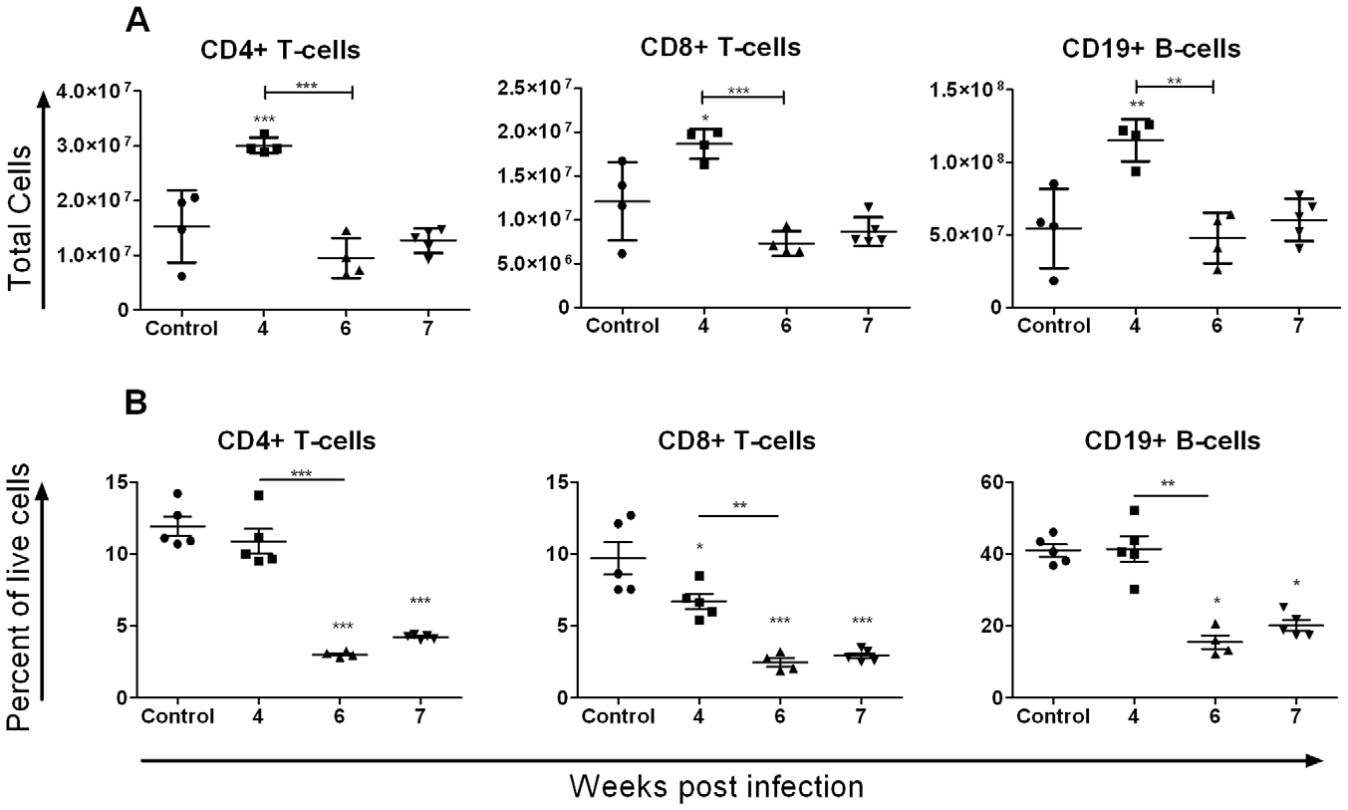
Flow cytometry revealed dynamic changes in lymphocyte populations of the spleen over time. A: Flow cytometry demonstrated a significant increase in the total number of CD4^+^ and CD8^+^ T-cells and CD19^+^ B-cells in the spleen at 4 weeks p.i. followed by return of these cells to baseline levels by 6 weeks p.i. B: The ratio of T- and B-cells to total live cells decreased significantly over time. Values represent means ±1SD. N = 5 per group except for 6 weeks p.i. where one mouse harboured no adult worms and was excluded from all further analyses.

Flow cytometry for CD8^+^ T-cells, CD4^+^ T-cells and B-cells revealed an early increase in the total number of these cells at 4 weeks p.i. followed by a return to baseline levels by 6 weeks p.i. (Figure 3A). In contrast, the ratio of T-cells and B-cells to total splenocytes decreased significantly after 4 weeks p.i. (Figure 3B).

### Microarray Analysis

#### Transcriptional profile of the spleen during *S. japonicum* infection - Filtering of microarray data

**Figure 4:**
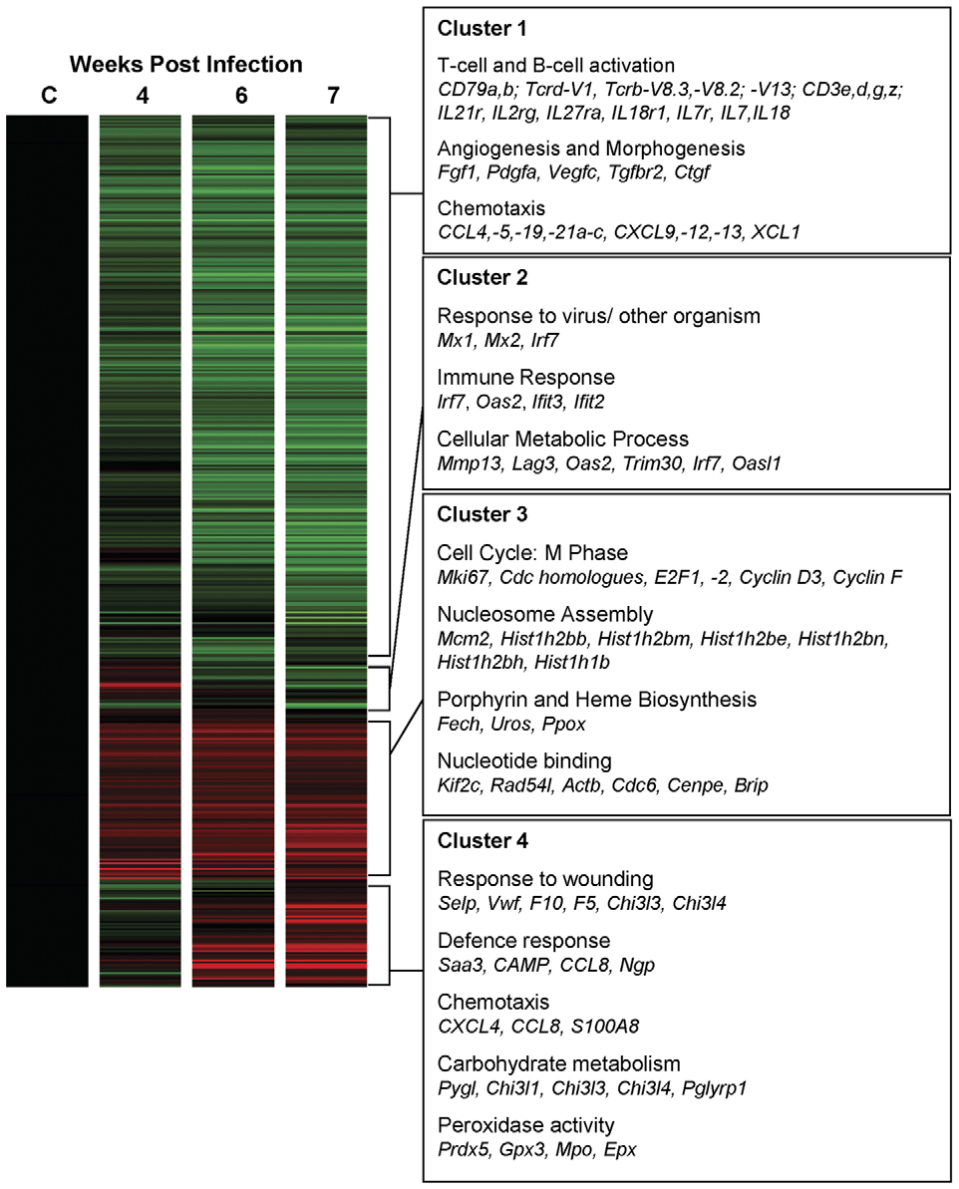
Hierarchical clustering and prominent gene ontologies of genes differentially expressed in the spleen. Four distinct clusters representing genes that were significantly down-regulated (cluster 1); up-regulated earlier (cluster 2), consistently up-regulated (cluster 3) and up-regulated later (cluster 4) were identified by hierarchical clustering analysis. Prominent biological processes and molecular functions (gene ontologies) associated with genes 2-fold or greater up- or down-regulated in each of these clusters and genes associated with these ontologies are listed in the boxed text. Data are represented in heat map form where green represents down-regulated gene expression, red represents up-regulated expression, with relatively unchanged expression coloured black.

Normalised data for each of the 46,643 genes on the microarray were filtered for significant signal, thereby reducing the data set to 18,438 genes of which 4,553 were shown to be differentially expressed (1-Way ANOVA, p≤0.05). Hierarchical clustering identified four distinct patterns of gene expression (Figure 4) comprised of genes (Dataset S1) that were:

- Down-regulated during infection (3045 genes)
- Up-regulated early in infection (180 genes)
- Consistently up-regulated during infection (806 genes)
- Up-regulated late in infection (478 genes)

Functional annotation clustering of genes 2-fold or greater up- or down-regulated revealed that each of these gene clusters was associated with distinct gene ontologies (Figure 4; Table S2). Accession numbers and Illumina Probe IDs of genes of interest are provided (Table S3).

There was significant and sustained down-regulation of genes associated with B-cell and T-cell activation and proliferation, including components of the B- and T-cell receptor complexes including immunoglobulin genes (*Igh-VJ558*, *Igh-VS107*), B-cell receptor accessory molecules (e.g. *CD79a,b*), T-cell receptor chains (*Tcrd-V1*, *Tcrb-V8.3*, *Tcrb-V8.2*, *Tcrb-V13*) and T-cell receptor accessory molecules (*CD3e,d,g,z*). Additionally, there was sustained down-regulation of several cytokines and cytokine receptors associated with lymphocyte activation (e.g. *IL21r*, *IL2rg*, *IL27ra*, *IL18r1*, *IL7r*, *IL7*, and *IL18*). Down-regulation of genes associated with chemotaxis, including numerous chemokines (*CCL4,-5,-19,-21a–c*, *CXCL9,-12,-13*, *XCL1*), was also a prominent feature of the splenic transcriptional response to schistosome infection. Genes involved in morphogenesis and angiogenesis (e.g. *Fgf1*, *Pdgfa*, *Vegfc*, *Ctgf*, *Tgfbr2*) were also down-regulated from 4 weeks p.i.

Genes consistently up-regulated were predominantly associated with progression through the cell cycle and included key cell proliferation markers (e.g. *Mki67*) and genes associated with the M-phase of the cell cycle (e.g. Cell division cycle homologues, *E2F1* and *2*, *Cyclin D3*, *Cyclin F*). Significant up-regulation of key enzymes of the porphyrin and heme biosynthesis pathways (e.g. *Fech*, *Uros*, *Ppox*) was also observed.

Genes up-regulated early in infection were functionally associated with immune responses including several interferon-inducible genes such as *Irf7*, *Oas2*, *Ifit3* and *Ifit2*. Genes up-regulated in the spleen later in infection were associated with wound healing and defence responses as well as chemotaxis (e.g. *CXCL4*, *CCL8*, *S100A8*), glycolysis and peroxidase activity. Other genes whose expression increased significantly over time included chitinase-like genes (e.g. *Chi3l1*, *Chi3l3*, *Chi3l4*), neutrophils markers (e.g. *Ngp*, *NE*), eosinophil markers (e.g. *Epx* and *Ear1–3,6,10*), Annexin a1 (*ANXA1*) and Cathelicidin Antimicrobial Peptide (*CAMP*).

#### Comparison of transcriptional profiles of the spleen and liver

Comparison of spleen and liver microarray datasets identified 297 genes that were commonly up-regulated and 78 genes that were commonly down-regulated. There was spleen specific up-regulation of 367 genes and specific down-regulation of 863 genes. Forty-one genes up-regulated in the spleen were down-regulated in the liver and 242 genes down-regulated in the spleen were concurrently up-regulated in the liver. There was liver specific up-regulation of 1,025 genes and down-regulation of 1,255 genes (Dataset S2; Table S4).

Up-regulation of the proliferation marker *Mki67* and genes associated with progression through the cell cycle was common to both liver and spleen (e.g. *Cyclin B*, *Cdc20*, *Cdca3*). Other commonly up-regulated genes included the chemokines *CCL8*, *S100A8*, *S100A9*, whose expression was higher in the liver [9]. Commonly down-regulated genes included some members of the Cytochrome P450 family (*Cyp27a1*, *Cyp2d22*, *Cyp4f13*, *Cyp4v3*) that have been associated with heme or iron binding, or mono-oxygenase activity.

#### Spleen specific responses

Elevated expression of genes encoding key enzymes of the heme and porphyrin biosynthesis pathways (e.g. *Fech*, *Uros*, *Ppox*) was specific to the spleen. Sustained down-regulation of cytokine (*IL7*, *IL18*) and cytokine receptor genes (e.g. *IL7r*, *IL18r*, *IL21r*, *IL27ra*) associated with lymphocyte activation was observed in the spleen while their expression was unchanged in the liver.

#### Liver specific responses

Elevated expression of several procollagen genes, including *COL1A1*, as well as several profibrotic cytokines (*TGF-β*, *PDGF-β*, *EDN1*) was observed exclusively in the liver [9]. Similarly, there was significant down-regulation of numerous components of several metabolic pathways including lipid, fatty acid and amino acid metabolism [9].

#### Contrasting expression of genes associated with cell recruitment

**Figure 5:**
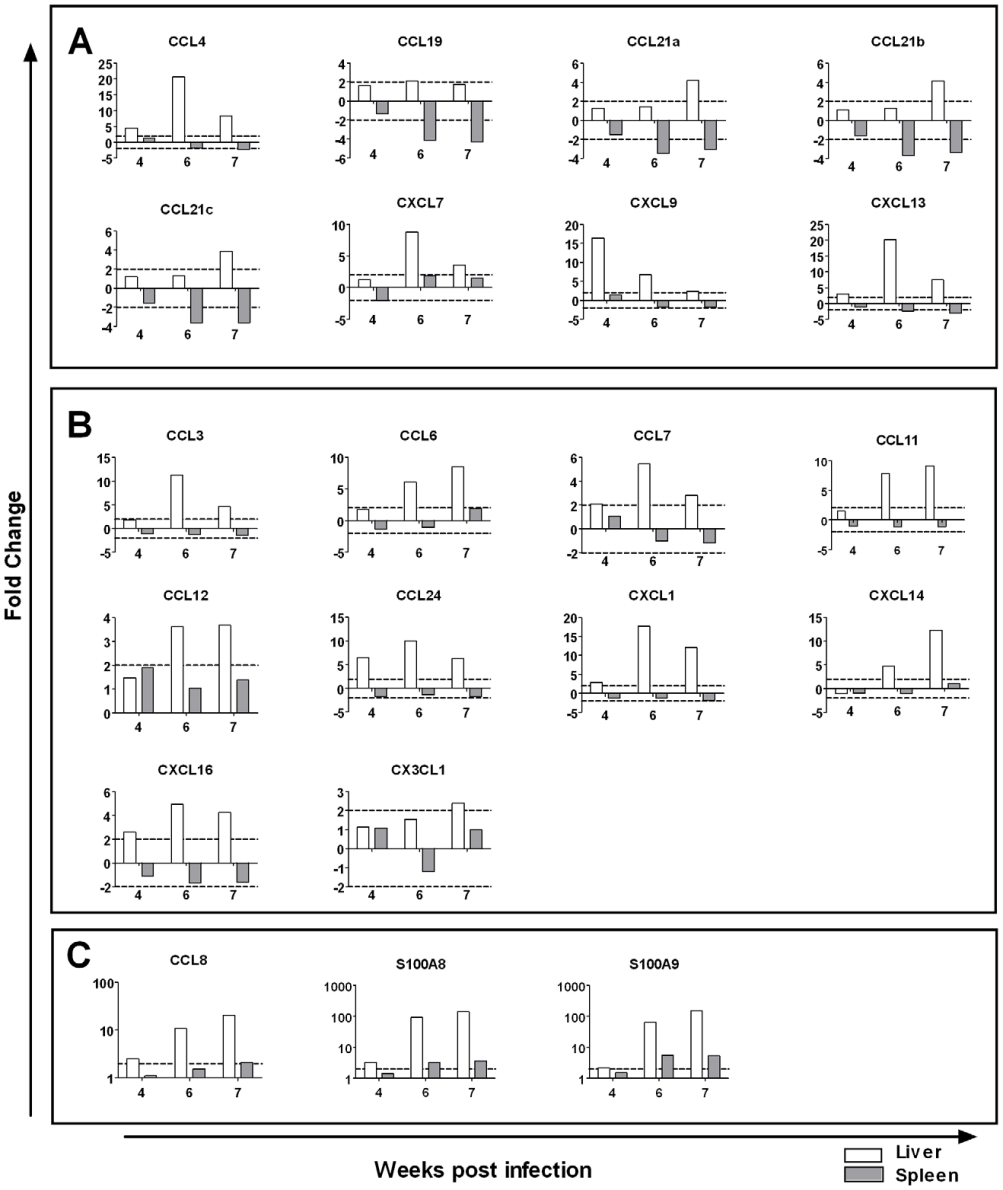
Contrasting expression of chemokines in the liver and spleen by microarray analysis. Comparison of spleen and liver transcriptional profiles identified a number of chemokines with contrasting expression. A: Chemokines up-regulated in the liver and down-regulated in the spleen during schistosome infection. B: Chemokines up-regulated in the liver and unchanged or below levels of detection in the spleen. C: Chemokines up-regulated in both organs showed greater expression in the liver. Graphs represent average fold change relative to uninfected tissue by microarray analysis. Dotted lines represent a ±2 fold cut-off for biological significance. Expression values for the liver are derived from our previous study of the transcriptional profile of the *S. japonicum* infected liver [9].

Distinct differences were observed in the expression of genes associated with chemotaxis in the liver and spleen (Figure 5). The chemokines *CCL4,-19,-21a-c*, *CXCL7,-9,-13* and the cell adhesion molecules *ICAM1*, *ICAM 2*, down-regulated in the spleen, were concurrently up-regulated in the liver [9]. Further, the chemokines *CCL3*, *-6*, *-7*, *-11*, *-12*, *-24*, *CXCL-1*, *-14*, *-16* and *CX3CL1* and the cell adhesion molecules *VCAM1*, *NCAM1*, *PECAM1* were up-regulated in the liver [9] while unchanged or undetectable in the spleen. Expression of chemokines (*CCL8*, *S100A8*, *S100A9*) up-regulated in both organs but was higher in the liver [9].

### Real-Time PCR

Real-time PCR was performed on a subset of genes representative of transcripts that were highly up- or down-regulated in the spleen (*NE*, *EPX*, *Chi3l3*, *CXCL13*); exhibited contrasting expression in the liver and spleen (*CXCL13*, *CXCL1*, *CXCL9*) and are key genes from important biological categories identified by DAVID analysis (Cell cycle: *Mki67*; Chemotaxis: *CXCL1*, *CXCL4*, *CXCL9*, *CXCL*13); as well as genes encoding Th1/Th2 cytokines (*IFN-γ*, *IL-4*). The results of the real-time PCR analyses correlated closely with those observed by microarray analysis (Spearman's correlation r = 0.93, p≤0.0001, n = 36) (Figure S1).

## Discussion

To date, there have been no studies conducted to specifically define the molecular or transcriptional processes occurring in the spleen during infection with any schistosome species. We have reported here the first such study employing microarray analysis in combination with flow cytometry and histochemistry to characterise the transcriptional and cellular profile of the spleen following schistosome infection, comparing these changes with those occurring in the liver [9].

Overall, the transcriptional response of the spleen reflected cellular changes in this organ during progression of *S. japonicum* infection. Significant up-regulation of proliferation markers and genes associated with the cell cycle and lymphocyte proliferation paralleled the expansion of T- and B-cells within the spleen. Similarly, the significant down-regulation of genes associated with T- and B-cell receptor signalling reflected the decrease in the relative proportion of these cells in the splenic compartments over time. The down-regulation of the cytokines that promote Th1 development from 6 weeks p.i. could favour the development of a Th2 response and reflects the shift from a Th1 to a Th2 dominant response observed in the liver at this time point [1], [8], [9]. Further, down-regulation of a large cohort of genes predominantly associated with immune responses is likely to reflect attempts by the host to regulate the immune response to schistosome infection.

Loss of B-cells/T-cells following initial expansion could be due to cell death or migration from the spleen. We did not detect elevated expression of apoptosis-associated genes in the spleen but there was decreased expression of several chemokines, especially lymphocyte chemokines such as CXCL13, CCL21 and CCL19. Combined with the accumulation of these cells in the liver [9], these results suggest that cellular loss from the spleen is more likely due to migration than apoptosis. Further, it has been shown that *S. mansoni* infections are able to influence hematopoietic processes occurring in the bone marrow as well as the activity of bone marrow-derived cells (e.g [22], [23]). This raises the alternative possibility that infection-induced modifications in haematopoiesis occurring up-stream of the spleen, contribute to the loss of B- and T-cells from the spleen during *S. japonicum* infection. Further studies to define the contribution of specific chemokines, apoptosis and alterations to the haematopoietic process within the bone marrow are required if we are to fully comprehend the mechanisms involved.

Accumulation of eosinophils, neutrophils and macrophages in the spleen was paralleled by enhanced expression of neutrophil and eosinophil markers as well as genes known to be expressed at high levels in these cells such as annexin A1 (*ANXA1*) and Cathelicidin antimicrobial peptide (*CAMP*). ANXA1 regulates polymorphonuclear leukocyte trafficking and function during innate immune responses and T-cell dependent inflammation during adaptive immune responses [24]. CAMP is known to have direct antimicrobial activity, is chemotactic for a variety of cells, and regulates production of pro-inflammatory cytokines [25]. The role of these genes in schistosomiasis has not been investigated but their increased expression in the spleen suggests that they may be involved in shaping the immune response to schistosome infections.

Significant up-regulation of *Chi3l3* and increased staining for the macrophage marker F4/80 suggests that there may be an increase in the population of alternatively activated macrophages in the spleen during schistosome infection. Similarly, there was significant induction of the alternatively activated macrophage markers *Retnla* and *Mrc1* and increases in the number of F4/80^+^ macrophages in the liver. In light of recent studies demonstrating immunoregulatory roles for alternatively activated macrophages during Th2 responses [26], [27], [28], it is possible that their presence in the liver and spleen during schistosome infection represents a mechanism whereby the host regulates the immune response to infection at both the local and systemic level.

Comparison of the transcriptional profiles of the liver and spleen revealed common up-regulation of genes associated with progression through the cell cycle indicating that cellular proliferation is occurring in both organs during infection. Up-regulation of components of the heme and porphyrin metabolism pathways was specific to the spleen and likely reflects increased blood volume passing through this organ associated with portal hypertension.

Up-regulation of extracellular matrix components, as well as the profibrotic cytokines *TGF-β*, *EDN1* and *PDGF-β*, was enhanced in the liver but remained unchanged in the spleen reflecting the development of fibrosis [9]. Similarly, there was liver specific down-regulation of many components of several metabolic pathways including, but not restricted to, metabolism of xenobiotics, bile acid biosynthesis, fatty acid metabolism and glutathione metabolism [9]. As discussed previously [9], these results are consistent with studies of the metabolic function of the liver following schistosome infection and are indicative of decreased liver function associated with hepatic injury [29], [30], [31].

The most striking difference between the liver and spleen was the contrasting expression of genes involved in cellular recruitment (Figure 5). Several chemokines, including lymphoid homing and T-cell chemokines (e.g. *CXCL13*, *CCL19*, *CCL21a–c*), were significantly up-regulated in the liver [9] but down-regulated in the spleen. Further, expression of eosinophil (*CCL24*), neutrophil (*CXCL1*) and macrophage/monocyte (*CCL6*, *CCL7*, *CXCL14*) chemokines and the cell adhesion molecules *VCAM1*, *NCAM1*, *PECAM1* was enhanced in the liver [9] but was unchanged or undetectable in the spleen. The differential expression of these genes likely contributes to the generation of a chemotactic signalling gradient promoting the recruitment of effector cells, including eosinophils, neutrophils and lymphocytes, to the liver during infection leading to the development of granulomas and fibrosis [9]. The observed loss of T- and B-cells from the spleen after initial expansion at 4 weeks p.i. is, therefore, more likely due to migration of these cells from this organ to the peripheral tissues than cell death. Similar induction of CCL21, CXCL16, CXCL9 and CXCL13 in the liver in other models of hepatic disease suggests that common mechanisms regulate the recruitment of lymphocytes to the liver following inflammation [32], [33], [34]. Down-regulation of lymphoid homing chemokines in the spleen has been implicated in alterations in lymphoid structure during *Leishmania* infection [2], altered motility of dendritic cells and lymphocytes following several viral infections [3], and with decreased responsiveness to secondary infection following primary infection with lymphocytic choriomeningitis virus (LCMV) or *Listeria monocytogenes* [3]. The down-regulation of these chemokines during *S. japonicum* infection may contribute to the significant changes observed in the spleen during schistosome infection and may go some way to explaining how schistosomes skew the immune response to other infections.

Comparison of the expression profiles of the liver and spleen clearly indicate that there is co-ordinated expression of chemokines in these organs during *S. japonicum* infection. Up-regulation of lymphocyte, eosinophil and monocyte chemokines in the liver [9], and down-regulation of the same chemokines in the spleen may contribute to the development of a chemotactic signalling gradient that promotes recruitment of these cells to the liver, thereby facilitating the development of granulomas and fibrosis. Furthermore, the down-regulation of homeostatic lymphoid chemokines, such as CXCL13 and CCL21, in the spleen could lead to disruption of the splenic architecture and the altered immune responses associated with schistosome infections. Additionally, we observed up-regulation of the alternatively activated macrophage marker Chi3l3 and the immunoregulatory molecules ANXA1 and CAMP in the spleen. These results suggest that the spleen may be an important site for the regulation of *S. japonicum*-induced immune responses. Together these data highlight the importance of the spleen to the immunopathogenesis of schistosomiasis and significantly enhance our understanding of the chemokine signalling pathways regulating the development of schistosome-induced granulomas and fibrosis.

## Supporting Information

Dataset S1 Gene lists generated by filtering and hierarchical clustering of microarray data from the spleen. Accession numbers, gene symbols and descriptions were not available for some genes.
(4.72 MB XLS)

Dataset S2 Gene lists generated by comparison of microarray data from the liver and spleen. - designates not detected. Accession numbers, gene symbols and descriptions were not available for some genes.
(1.03 MB XLS)

Figure S1 Real-time PCR correlated well with microarray results. Real-time PCR on a subset of genes expressed in the spleen correlated well with the results of microarray analyses (Spearman's correlation r = 0.93, p<0.0001, n = 36). Expression of genes analysed by real-time PCR is depicted in the line graphs and is displayed as mean fold change ±1SD relative to uninfected controls. Colour bars are representative of corresponding microarray data where down-regulation is coloured green, up-regulated expression is coloured red and relatively unchanged expression is coloured black. *p≤0.05, **p≤0.01, ***p≤0.001 in comparison to uninfected spleen unless otherwise indicated.
(0.27 MB TIF)

Table S1 Primers used for real-time PCR confirmation of microarray results. Primer source: 1. Chiu B-C, et al.(2003) Am J Respir Cell Mol Biol 29: 106–116. 2. Hesse M, et al. (2004) J Immunol 172: 3157–3166. 3. Amante FH, et al. (2007) Am J Pathol 171: 548–559.
(0.04 MB DOC)

Table S2 Top five functional annotation groups of spleen hierarchical clusters. The top five functional annotation groups and unique biological processes/molecular functions with statistical significance for each spleen hierarchical cluster are listed (p≤0.05). Functional annotation groups are ranked on the basis of enrichment score biological processes/molecular functions within these groups are ranked on the basis of their p-value (Modified Fischer's exact test, EASE score).
(0.14 MB DOC)

Table S3 Accession numbers and Illumina Probe IDs of genes of interest. Accession numbers and descriptions were not available for some genes.
(0.12 MB DOC)

Table S4 Key genes and functional categories showing differential expression in the liver and spleen. ^+^Expression values were generated from microarray data and are displayed as a ratio relative to un-infected mice; *Expression values represent the mean of two or more probes. ^#^Expression values for the liver are derived from our previous study of the transcriptional profile of the *S. japonicum* infected liver (Burke et al. 2009 PLoS NTD, In Press). - not detected.
(0.38 MB DOC)
