# Supplementary figures and images for: Co-ordinated Gene Expression in the Liver and Spleen during Schistosoma japonicum Infection Regulates Cell Migration
Source: PLoS Negl Trop Dis. 2010 May 18;4(5):e686. doi: 10.1371/journal.pntd.0000686 (PMC2872641; doi:10.1371/journal.pntd.0000686)

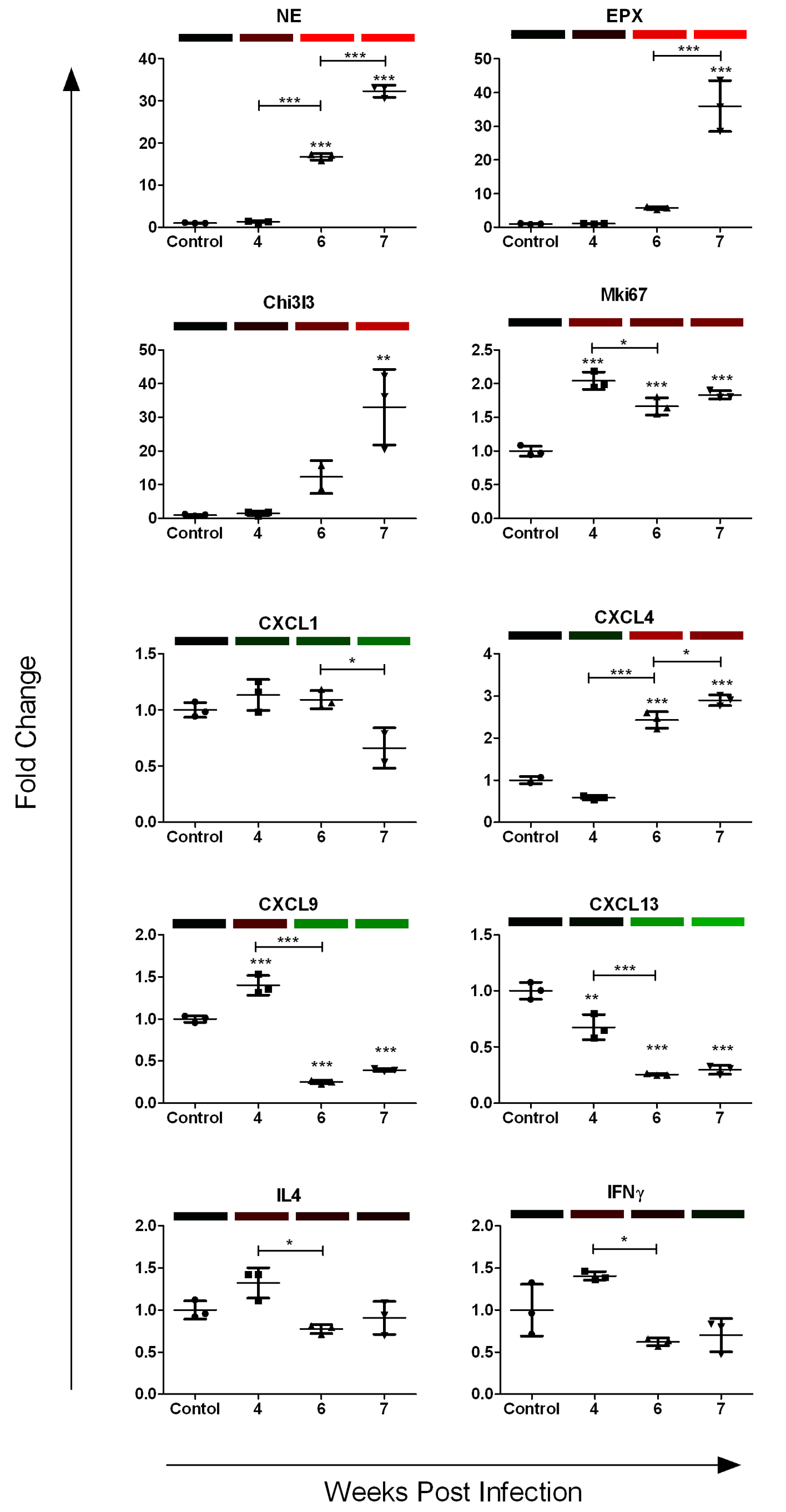

Supplement: Figure S1 — Real-time PCR correlated well with microarray results. Real-time PCR on a subset of genes expressed in the spleen correlated well with the results of microarray analyses (Spearman's correlation r = 0.93, p<0.0001, n = 36). Expression of genes analysed by real-time PCR is depicted in the line graphs and is displayed as mean fold change ±1SD relative to uninfected controls. Colour bars are representative of corresponding microarray data where down-regulation is coloured green, up-regulated expression is coloured red and relatively unchanged expression is coloured black. *p≤0.05, **p≤0.01, ***p≤0.001 in comparison to uninfected spleen unless otherwise indicated. (0.27 MB TIF) [file pntd.0000686.s003.tif]
