# Supplementary material for: Co-ordinated Gene Expression in the Liver and Spleen during Schistosoma japonicum Infection Regulates Cell Migration
Source: PLoS Negl Trop Dis. 2010 May 18;4(5):e686. doi: 10.1371/journal.pntd.0000686 (PMC2872641; doi:10.1371/journal.pntd.0000686)
Supplement: Table S1 — Primers used for real-time PCR confirmation of microarray results. Primer source: 1. Chiu B-C, et al.(2003) Am J Respir Cell Mol Biol 29: 106–116. 2. Hesse M, et al. (2004) J Immunol 172: 3157–3166. 3. Amante FH, et al. (2007) Am J Pathol 171: 548–559. (0.04 MB DOC) [file pntd.0000686.s004.doc]

| **Gene Name** | **Forward Primer** | **Reverse Primer** | **Amplicon Length (bp)** | **Primer Source** |
| --- | --- | --- | --- | --- |
| *Chi3l3* | 5' gtt ttt cca cag cgc att ct | 5' tgg tgg ttt tac agg aag ca | 199 | Primer-Blast |
| *NE* | 5' gtg gtg act aac atg tgc cg | 5' cga agg cat ctg ggt aca at | 175 | Primer-Blast |
| *EPX* | 5' ggt ttc gag gga cat ctt ca | 5' ccc gtt acc ctc atc ttc aa | 155 | Primer-Blast |
| *CXCL4* | 5' gag ccc tag acc cat ttc ct | 5' gat ctc cat cgc ttt ctt cg | 187 | Primer-Blast |
| *Mki67* | 5' cag tac tcg gaa tgc agc aa | 5' cag tct tca ggg gct ctg tc | 170 | Primer-Blast |
| *CXCL9* | 5' ctg agg ctc acg tca cca agt c | 5' ggc tct agg ctg acc caa atg | 110 | 1 |
| *CXCL1* | 5' gct ggg att cac ctc aag aa | 5' tct ccg tta ctt ggg gac ac | 180 | Primer-Blast |
| *IL4* | 5' acg agg tca cag gag aag gga | 5' agc cct aca gac gag ctc act c | 101 | 2 |
| *IL13* | 5' ggc agc atg gta tgg agt gtg | 5' tgg gtc ctg tag atg gca ttg | 101 | 2 |
| *IFNG* | 5' cac ggc aca gtc att gaa ag | 5' gct gat ggc ctg att gtc tt | 198 | Primer-Blast |
| *HPRT* | 5' gtt gga tac agg cca gac ttt gtt g | 5' gat tca acc ttg cgc tca tct tag gc | 163 | 3 |
