# Supplementary material for: Co-ordinated Gene Expression in the Liver and Spleen during Schistosoma japonicum Infection Regulates Cell Migration
Source: PLoS Negl Trop Dis. 2010 May 18;4(5):e686. doi: 10.1371/journal.pntd.0000686 (PMC2872641; doi:10.1371/journal.pntd.0000686)
Supplement: Table S2 — Top five functional annotation groups of spleen hierarchical clusters. The top five functional annotation groups and unique biological processes/molecular functions with statistical significance for each spleen hierarchical cluster are listed (p≤0.05). Functional annotation groups are ranked on the basis of enrichment score biological processes/molecular functions within these groups are ranked on the basis of their p-value (Modified Fischer's exact test, EASE score). (0.14 MB DOC) [file pntd.0000686.s005.doc]

| **Gene Cluster** | **Functional Annotation Group** | **Biological Process/Molecular Function** | **p-value** | **Enrichment  Score** |
| --- | --- | --- | --- | --- |
| 1 | 1 | GO:0046649~lymphocyte activation | 4.15E-18 | 7.99 |
|  |  | GO:0045321~leukocyte activation | 5.87E-17 |  |
|  |  | GO:0002376~immune system process | 7.27E-17 |  |
|  |  | GO:0001775~cell activation | 1.22E-15 |  |
|  |  | GO:0042110~T cell activation | 3.95E-14 |  |
|  |  |  |  |  |
|  | 2 | GO:0050865~regulation of cell activation | 1.61E-10 | 5.24 |
|  |  | GO:0051249~regulation of lymphocyte activation | 5.00E-10 |  |
|  |  | GO:0050863~regulation of T cell activation | 6.10E-10 |  |
|  |  | GO:0051251~positive regulation of lymphocyte activation | 6.41E-08 |  |
|  |  | GO:0046651~lymphocyte proliferation | 6.60E-06 |  |
|  |  |  |  |  |
|  | 3 | GO:0001568~blood vessel development | 1.08E-06 | 4.48 |
|  |  | GO:0001944~vasculature development | 1.38E-06 |  |
|  |  | GO:0048514~blood vessel morphogenesis | 1.59E-06 |  |
|  |  | GO:0048514~blood vessel morphogenesis | 2.29E-06 |  |
|  |  | GO:0001525~angiogenesis | 3.01E-06 |  |
|  |  |  |  |  |
|  | 4 | GO:0030246~carbohydrate binding | 1.69E-05 | 3.75 |
|  |  | GO:0005529~sugar binding | 5.87E-04 |  |
|  |  | *No other significant ontologies* |  |  |
|  |  |  |  |  |
|  | 5 | GO:0005096~GTPase activator activity | 2.29E-04 | 2.95 |
|  |  | GO:0008047~enzyme activator activity | 3.01E-04 |  |
|  |  | *No other significant ontologies* |  |  |
|  |  |  |  |  |
| 2 | 1 | GO:0009615~response to virus | 8.74E-04 | 2.51 |
|  |  | GO:0009615~response to virus | 9.75E-04 |  |
|  |  | GO:0051707~response to other organism | 6.09E-03 |  |
|  |  | GO:0051707~response to other organism | 8.36E-03 |  |
|  |  | GO:0051704~multi-organism process | 1.67E-02 |  |
|  |  | GO:0009607~response to biotic stimulus | 1.86E-02 |  |
|  |  |  |  |  |
|  | 2 | GO:0006955~immune response | 2.85E-08 | 1.92 |
|  |  | GO:0002376~immune system process | 7.20E-07 |  |
|  |  | GO:0050896~response to stimulus | 1.02E-04 |  |
|  |  | GO:0016779~nucleotidyltransferase activity | 6.29E-03 |  |
|  |  | *No other significant ontologies* |  |  |
|  |  |  |  |  |
|  |  | *No other significant annotation clusters* |  |  |
|  |  |  |  |  |
| 3 | 1 | GO:0051301~cell division | 1.54E-11 | 8.77 |
|  |  | GO:0007049~cell cycle | 3.12E-11 |  |
|  |  | GO:0000279~M phase | 1.12E-10 |  |
|  |  | GO:0007067~mitosis | 1.25E-10 |  |
|  |  | GO:0000087~M phase of mitotic cell cycle | 1.36E-10 |  |
|  |  |  |  |  |
|  | 2 | GO:0006334~nucleosome assembly | 3.49E-08 | 5.07 |
|  |  | GO:0065004~protein-DNA complex assembly | 1.13E-07 |  |
|  |  | GO:0031497~chromatin assembly | 1.68E-07 |  |
|  |  | GO:0006259~DNA metabolic process | 8.30E-07 |  |
|  |  | GO:0051276~chromosome organization and biogenesis | 2.79E-06 |  |
|  |  |  |  |  |
|  | 3 | GO:0006996~organelle organization and biogenesis | 2.27E-06 | 4.39 |
|  |  | GO:0051276~chromosome organization and biogenesis | 2.79E-06 |  |
|  |  | GO:0016043~cellular component organization and biogenesis | 1.04E-02 |  |
|  |  | *No other significant ontologies* |  |  |
|  |  |  |  |  |
|  | 4 | GO:0046148~pigment biosynthetic process | 3.20E-04 | 2.66 |
|  |  | GO:0006783~heme biosynthetic process | 3.82E-04 |  |
|  |  | GO:0042440~pigment metabolic process | 5.46E-04 |  |
|  |  | GO:0033014~tetrapyrrole biosynthetic process | 5.98E-04 |  |
|  |  | GO:0006779~porphyrin biosynthetic process | 5.98E-04 |  |
|  |  |  |  |  |
|  | 5 | GO:0007059~chromosome segregation | 3.99E-04 | 2.17 |
|  |  | GO:0000819~sister chromatid segregation | 2.72E-02 |  |
|  |  | *No other significant ontologies* |  |  |
|  |  |  |  |  |
| 4 | 1 | GO:0009605~response to external stimulus | 3.81E-13 | 7.01 |
|  |  | GO:0009611~response to wounding | 4.09E-10 |  |
|  |  | GO:0007596~blood coagulation | 1.27E-08 |  |
|  |  | GO:0050817~coagulation | 1.40E-08 |  |
|  |  | GO:0007599~hemostasis | 1.97E-08 |  |
|  |  |  |  |  |
|  | 2 | GO:0009611~response to wounding | 4.09E-10 | 4.92 |
|  |  | GO:0006950~response to stress | 2.13E-07 |  |
|  |  | GO:0006954~inflammatory response | 4.05E-04 |  |
|  |  | *No other significant ontologies* |  |  |
|  |  |  |  |  |
|  | 3 | GO:0006952~defense response | 7.91E-08 | 2.76 |
|  |  | GO:0051707~response to other organism | 1.47E-03 |  |
|  |  | GO:0042742~defense response to bacterium | 2.27E-03 |  |
|  |  | GO:0051704~multi-organism process | 1.02E-02 |  |
|  |  | GO:0009607~response to biotic stimulus | 1.12E-02 |  |
|  |  |  |  |  |
|  | 4 | GO:0006935~chemotaxis | 2.00E-03 | 2.21 |
|  |  | GO:0042330~taxis | 2.00E-03 |  |
|  |  | GO:0042221~response to chemical stimulus | 1.52E-02 |  |
|  |  | GO:0007626~locomotory behavior | 2.28E-02 |  |
|  |  | GO:0007610~behavior | 3.77E-02 |  |
|  |  |  |  |  |
|  | 5 | GO:0042744~hydrogen peroxide catabolic process | 1.03E-03 | 2.17 |
|  |  | GO:0042743~hydrogen peroxide metabolic process | 2.42E-03 |  |
|  |  | GO:0006800~oxygen and reactive oxygen species metabolic process | 3.48E-03 |  |
|  |  | GO:0042542~response to hydrogen peroxide | 3.72E-03 |  |
|  |  | GO:0004601~peroxidase activity | 6.48E-03 |  |
