# Supplementary material for: Co-ordinated Gene Expression in the Liver and Spleen during Schistosoma japonicum Infection Regulates Cell Migration
Source: PLoS Negl Trop Dis. 2010 May 18;4(5):e686. doi: 10.1371/journal.pntd.0000686 (PMC2872641; doi:10.1371/journal.pntd.0000686)
Supplement: Table S3 — Accession numbers and Illumina Probe IDs of genes of interest. Accession numbers and descriptions were not available for some genes. (0.12 MB DOC) [file pntd.0000686.s006.doc]

| **Gene Symbol** | **Illumina Probe ID** | **Description** | **Accession Number** |
| --- | --- | --- | --- |
| *ANXA1* | 2320053 | annexin A1 | NM_010730 |
| *CAMP* | 6290735 | cathelicidin antimicrobial peptide | NM_009921 |
| *CCL3* | 2810092 | chemokine (C-C motif) ligand 3 | NM_011337 |
| *CCL4* | 50368 | chemokine (C-C motif) ligand 4 | NM_013652 |
| *CCL4* | 430047 | chemokine (C-C motif) ligand 4 | NM_013652 |
| *CCL5* | 3710397 | chemokine (C-C motif) ligand 5 | NM_013653 |
| *CCL6* | 6660390 | chemokine (C-C motif) ligand 6 | NM_009139 |
| *CCL7* | 2650519 | chemokine (C-C motif) ligand 7 | NM_013654 |
| *CCL8* | 3870010 | chemokine (C-C motif) ligand 8 | NM_021443 |
| *CCL11* | 1770347 | small chemokine (C-C motif) ligand 11 | NM_011330 |
| *CCL12* | 2120746 | chemokine (C-C motif) ligand 12 | NM_011331 |
| *CCL12* | 7000592 | chemokine (C-C motif) ligand 12 | NM_011331 |
| *CCL19* | 5080487 | chemokine (C-C motif) ligand 19 | NM_011888 |
| *CCL21a* | 5050338 | chemokine (C-C motif) ligand 21a | NM_011335 |
| *CCL21b* | 4670176 | chemokine (C-C motif) ligand 21b | NM_011124 |
| *CCL21c* | 6770037 | chemokine (C-C motif) ligand 21c | NM_023052 |
| *CCL24* | 670129 | chemokine (C-C motif) ligand 24 | NM_019577 |
| Ccnb1 | 4780372 | cyclin B1 | NM_172301 |
| Ccnd3 | 380528 | cyclin D3 | NM_007632 |
| Ccnf | 6370288 | cyclin F | NM_007634 |
| *CD3d* | 2810739 | CD3 antigen, delta polypeptide | NM_013487 |
| *CD3e* | 3800056 | CD3 antigen, epsilon polypeptide | NM_007648 |
| *CD3g* | 2680288 | CD3 antigen, gamma polypeptide (Cd3g), mRNA. | NM_009850 |
| *CD3z* | 5720136 | CD3 antigen, zeta polypeptide | NM_031162 |
| *CD79a* | 3450563 | CD79A antigen (immunoglobulin-associated alpha) | NM_007655 |
| *CD79b* | 1450066 | CD79B antigen | NM_008339 |
| *CD79b* | 3390358 | CD79B antigen | NM_008339 |
| *Cdc20* | 3440017 | cell division cycle 20 homolog (S. cerevisiae) | NM_023223 |
| *Cdc20* | 6220088 | cell division cycle 20 homolog (S. cerevisiae) | NM_023223 |
| *Cdc20* | 3440044 | cell division cycle 20 homolog (S. cerevisiae) | NM_023223 |
| *Cdca3* | 3940397 | cell division cycle associated 3 | NM_013538 |
| *Chi3l1* | 1170039 | chitinase 3-like 1 | NM_007695 |
| *Chi3l3* | 2120332 | chitinase 3-like 3 | NM_009892 |
| *Chi3l4* | 130487 | chitinase 3-like 4 | NM_145126 |
| *Chi3l4* | 103180682 | chitinase 3-like 4 | NM_145126 |
| *COL1A1* | 730020 | procollagen, type I, alpha 1 | NM_007742 |
| *Ctgf* | 4540577 | connective tissue growth factor | NM_010217 |
| *CX3CL1* | 3990707 | chemokine (C-X3-C motif) ligand 1 | NM_009142 |
| *CXCL1* | 2690537 | chemokine (C-X-C motif) ligand 1 | NM_008176 |
| *CXCL4* | 6130332 | chemokine (C-X-C motif) ligand 4 | NM_019932 |
| *CXCL7* | 5130446 | chemokine (C-X-C motif) ligand 7 | NM_023785 |
| *CXCL9* | 105860594 | chemokine (C-X-C motif) ligand 9 | NM_008599 |
| *CXCL9* | 1570673 | chemokine (C-X-C motif) ligand 9 | NM_008599 |
| *CXCL12* | 4570068 | chemokine (C-X-C motif) ligand 12, transcript variant 2 | NM_021704 |
| *CXCL12* | 4150750 | chemokine (C-X-C motif) ligand 12, transcript variant 2 | NM_013655 |
| *CXCL13* | 6290402 | chemokine (C-X-C motif) ligand 13 | NM_018866 |
| *CXCL14* | 6450324 | chemokine (C-X-C motif) ligand 14 | NM_019568 |
| *CXCL14* | 840114 | chemokine (C-X-C motif) ligand 14 | NM_019568 |
| *CXCL16* | 510278 | chemokine (C-X-C motif) ligand 16 | NM_023158 |
| *Cyp2d22* | 520750 | cytochrome P450, family 2, subfamily d, polypeptide 22 | NM_019823 |
| *Cyp4f13* | 3170132 | cytochrome P450, family 4, subfamily f, polypeptide 13 | NM_130882 |
| *Cyp4v3* | 5550390 | cytochrome P450, family 4, subfamily v, polypeptide 3 | NM_133969 |
| *Cyp27a1* | 5220035 | cytochrome P450, family 27, subfamily a, polypeptide 1 | NM_024264 |
| *E2F1* | 5360093 | E2F transcription factor 1 | NM_007891 |
| *E2F2* | 5270609 | E2F transcription factor 2 | NM_177733 |
| *E2F2* | 7000465 | E2F transcription factor 2 | NM_183301 |
| *Ear1* | 2360471 | eosinophil-associated, ribonuclease A family, member 1 | NM_007894 |
| *Ear2* | 106620332 | eosinophil-associated, ribonuclease A family, member 2 | NM_007895 |
| *Ear2* | 1340239 | eosinophil-associated, ribonuclease A family, member 2 | NM_007895 |
| *Ear2* | 5670239 | eosinophil-associated, ribonuclease A family, member 2 | NM_007895 |
| *Ear3* | 4120528 | eosinophil-associated, ribonuclease A family, member 3 | NM_017388 |
| *Ear3* | 104210204 | eosinophil-associated, ribonuclease A family, member 3 | NM_017388 |
| *Ear6* | 100580300 | eosinophil-associated, ribonuclease A family, member 6 | NM_053111 |
| *Ear6* | 3450670 | eosinophil-associated, ribonuclease A family, member 6 | NM_053111 |
| *Ear10* | 101230129 | eosinophil-associated, ribonuclease A family, member 10 | NM_053112 |
| *EDN1* | 1770047 | endothelin 1 | NM_010104 |
| *Epx* | 940022 | eosinophil peroxidase | NM_007946 |
| *Fech* | 2810605 | ferrochelatase | NM_007998 |
| *Fech* | 3120088 | ferrochelatase | NM_007998 |
| *Fgf1* | 5670601 | fibroblast growth factor 1 | NM_010197 |
| *Fgf1* | 4780435 | fibroblast growth factor 1 | NM_010197 |
| *ICAM1* | 6980138 | intercellular adhesion molecule | NM_010493 |
| *ICAM2* | 6400113 | intercellular adhesion molecule 2 | NM_010494 |
| *Ifit3* | 6200113 | interferon-induced protein with tetratricopeptide repeats 3 | NM_010501 |
| *Ifit3* | 2450278 | interferon-induced protein with tetratricopeptide repeats 3 | NM_010501 |
| *Igh-VJ558* | 60025 |  | XM_354700 |
| *Igh-VS107* | 460288 | immunoglobulin heavy chain (S107 family) | XM_354717 |
| *IL2rg* | 4120273 | interleukin 2 receptor, gamma chain | NM_013563 |
| *IL2rg* | 105700301 | interleukin 2 receptor, gamma chain | AK037584 |
| *IL7* | 5360440 | interleukin 7 | NM_008371 |
| *IL7r* | 100870368 | interleukin 7 receptor | AK041838 |
| *IL7r* | 103780026 | interleukin 7 receptor | AK040740 |
| *IL7r* | 6380500 | interleukin 7 receptor | NM_008372 |
| *IL18* | 107040095 | interleukin 18 | AK078100 |
| *IL18* | 6180039 | interleukin 18 | NM_008360 |
| *IL18r1* | 270546 | interleukin 18 receptor 1 | NM_008365 |
| *IL21r* | 7000273 | interleukin 21 receptor | NM_021887 |
| *IL27ra* | 940093 | interleukin 27 receptor, alpha | NM_016671 |
| *Irf7* | 1570605 | interferon regulatory factor 7 | NM_016850 |
| *Mki67* | 7050288 | antigen identified by monoclonal antibody Ki 67 | XM_133912 |
| *Mki67* | 3440750 | antigen identified by monoclonal antibody Ki 67 | XM_133912 |
| *NCAM1* | 105690114 | neural cell adhesion molecule 1 | NM_010875 |
| *NE* | 1570452 | neutrophil elastase | NM_015779 |
| *Ngp* | 2640133 | neutrophilic granule protein | NM_008694 |
| *Oas2* | 1230408 | 2-5 oligoadenylate synthetase 2 | NM_145227 |
| *Pdgfa* | 6020095 | platelet derived growth factor, alpha | NM_008808 |
| *Pdgfa* | 102630132 | platelet derived growth factor, alpha | NM_008808 |
| *PDGF-β* | 3060440 | platelet derived growth factor, B polypeptide | NM_011057 |
| *PECAM1* | 104480075 |  | NM_008816 |
| *PECAM1* | 4810139 |  | NM_008816 |
| *Ppox* | 2640678 | protoporphyrinogen oxidase | NM_008911 |
| *Ppox* | 3850102 | protoporphyrinogen oxidase | NM_008911 |
| *S100A8* | 70112 | S100 calcium binding protein A8 (calgranulin A) | NM_013650 |
| *S100A9* | 7050528 | S100 calcium binding protein A9 (calgranulin B) | NM_009114 |
| *Tcrb-V13* | 103800086 |  |  |
| *Tcrb-V8.2* | 3610048 |  |  |
| *Tcrb-V8.2* | 3520575 |  |  |
| *Tcrb-V8.3* | 105130594 |  |  |
| *Tcrd-V1* | 103990403 |  | AK045683 |
| *TGF-β* | 1940162 | transforming growth factor, beta 1 | NM_011577 |
| *Tgfbr2* | 1980537 | transforming growth factor, beta receptor II, transcript variant 1 | NM_009371 |
| *Uros* | 4120133 | uroporphyrinogen III synthase | NM_009479 |
| *Uros* | 3360139 | uroporphyrinogen III synthase | NM_009479 |
| *VCAM1* | 2900450 | vascular cell adhesion molecule 1 | NM_011693 |
| *VCAM1* | 101580538 | vascular cell adhesion molecule 1 | AK030195 |
| *Vegfc* | 5910494 | vascular endothelial growth factor C | NM_009506 |
| *XCL1* | 3800504 | chemokine (C motif) ligand 1 | NM_008510 |
