# Supplementary material for: Co-ordinated Gene Expression in the Liver and Spleen during Schistosoma japonicum Infection Regulates Cell Migration
Source: PLoS Negl Trop Dis. 2010 May 18;4(5):e686. doi: 10.1371/journal.pntd.0000686 (PMC2872641; doi:10.1371/journal.pntd.0000686)
Supplement: Table S4 — Key genes and functional categories showing differential expression in the liver and spleen. +Expression values were generated from microarray data and are displayed as a ratio relative to un-infected mice; *Expression values represent the mean of two or more probes. #Expression values for the liver are derived from our previous study of the transcriptional profile of the S. japonicum infected liver (Burke et al. 2009 PLoS NTD, In Press). - not detected. (0.38 MB DOC) [file pntd.0000686.s007.doc]

| **Functional Category/Gene Name** | **Accession Number** | **Symbol** | **Weeks Post Infection^+^** | | | | | | |
| --- | --- | --- | --- | --- | --- | --- | --- | --- | --- |
|  |  |  |  | **Spleen** |  |  |  | **Liver^#^** |  |
|  |  |  | **4** | **6** | **7** |  | **4** | **6** | **7** |
| ***Common up*** |  |  |  |  |  |  |  |  |  |
| **Cell Cycle** |  |  |  |  |  |  |  |  |  |
| cyclin B1 | NM_172301 | Ccnb1 | 2.1 | 2.4 | 1.9 |  | 1.2 | 2.6 | 2.0 |
| cell division cycle 20 homolog* | NM_023223 | Cdc20 | 1.6 | 2.1 | 1.8 |  | 1.6 | 2.7 | 2.2 |
| cell division cycle associated 2 | NM_175384 | Cdca2 | 1.8 | 2.2 | 1.8 |  | 1.8 | 3.4 | 3.2 |
| cell division cycle associated 3 | NM_013538 | Cdca3 | 1.7 | 2.0 | 2.1 |  | 2.9 | 6.5 | 5.9 |
| cell division cycle associated 8 | NM_026560 | Cdca8 | 1.6 | 2.1 | 1.8 |  | 1.2 | 2.1 | 1.8 |
| cyclin-dependent kinase inhibitor 3* | XM_484366 | Cdkn3 | 1.9 | 2.2 | 2.0 |  | 1.8 | 4.7 | 4.7 |
| E2F transcription factor 1 | NM_007891 | E2f1 | 1.7 | 2.2 | 1.9 |  | 1.1 | 2.2 | 1.8 |
| E2F transcription factor 2 | NM_177733 | E2f2 | 2.4 | 2.7 | 3.2 |  | 1.3 | 2.4 | 3.9 |
| minichromosome maintenance deficient 2 mitotin* | NM_008564 | Mcm2 | 1.8 | 2.2 | 1.7 |  | 1.4 | 3.3 | 2.9 |
| antigen identified by monoclonal antibody Ki 67* | XM_133912 | Mki67 | 2.0 | 2.1 | 2.2 |  | 2.2 | 5.5 | 6.3 |
|  |  |  |  |  |  |  |  |  |  |
| **Inflammatory response** |  |  |  |  |  |  |  |  |  |
| acyloxyacyl hydrolase | NM_012054 | Aoah | 1.3 | 2.4 | 2.8 |  | 2.0 | 4.9 | 3.9 |
| chemokine (C-C motif) ligand 8 | NM_021443 | Ccl8 | 1.1 | 1.5 | 2.1 |  | 2.5 | 10.8 | 20.2 |
| chitinase 3-like 4* | NM_145126 | Chi3l4 | 1.5 | 15.1 | 31.3 |  | 1.3 | 26.7 | 53.7 |
| integrin beta 2-like* | NM_008405 | Itgb2l | 1.2 | 6.2 | 15.2 |  | 1.1 | 2.2 | 9.7 |
| serum amyloid A 3 | NM_011315 | Saa3 | 1.1 | 6.9 | 14.6 |  | 9.9 | 103.8 | 26.3 |
| selectin, platelet | NM_011347 | Selp | 0.5 | 2.9 | 2.5 |  | 2.0 | 5.1 | 3.4 |
|  |  |  |  |  |  |  |  |  |  |
| **Chemotaxis** |  |  |  |  |  |  |  |  |  |
| chemokine (C-C motif) ligand 8 | NM_021443 | Ccl8 | 1.1 | 1.5 | 2.1 |  | 2.5 | 10.8 | 20.2 |
| interleukin 8 receptor, beta | NM_009909 | IL8rb | 0.6 | 0.9 | 2.3 |  | 1.0 | 2.5 | 3.9 |
| S100 calcium binding protein A8 (calgranulin A) | NM_013650 | S100a8 | 1.4 | 3.2 | 3.6 |  | 3.3 | 90.8 | 141.8 |
| S100 calcium binding protein A9 (calgranulin B) | NM_009114 | S100a9 | 1.5 | 5.5 | 5.2 |  | 2.1 | 62.2 | 153.0 |
|  |  |  |  |  |  |  |  |  |  |
| ***Common Down*** |  |  |  |  |  |  |  |  |  |
| **Monoxygenase and catalytic activity** |  |  |  |  |  |  |  |  |  |
| cytochrome P450, family 2, subfamily d, polypeptide 22* | NM_019823 | Cyp2d22 | 0.4 | 0.4 | 0.6 |  | 0.7 | 0.5 | 0.5 |
| cytochrome P450, family 4, subfamily f, polypeptide 13 | NM_130882 | Cyp4f13 | 0.6 | 0.4 | 0.6 |  | 0.8 | 0.4 | 0.5 |
| cytochrome P450, family 4, subfamily v, polypeptide 3 | NM_133969 | Cyp4v3 | 0.7 | 0.5 | 0.4 |  | 0.5 | 0.3 | 0.2 |
| kynurenine 3-monooxygenase (kynurenine 3-hydroxylase)* | NM_133809 | Kmo | 0.9 | 0.4 | 0.3 |  | 0.9 | 0.5 | 0.4 |
|  |  |  |  |  |  |  |  |  |  |
| ***Enhanced in the liver*** |  |  |  |  |  |  |  |  |  |
| **Chemokine/Cytokine activity** |  |  |  |  |  |  |  |  |  |
| chemokine (C-C motif) ligand 3 | NM_011337 | Ccl3 | - | - | - |  | 1.8 | 11.3 | 4.7 |
| chemokine (C-C motif) ligand 4* | NM_013652 | Ccl4 | 1.2 | 0.6 | 0.6 |  | 3.1 | 12.7 | 5.3 |
| chemokine (C-C motif) ligand 6 | NM_009139 | Ccl6 | 0.7 | 0.9 | 1.9 |  | 1.7 | 6.1 | 8.5 |
| small chemokine (C-C motif) ligand 11* | NM_011330 | Ccl11 | - | - | - |  | 1.5 | 7.8 | 9.1 |
| chemokine (C-C motif) ligand 12 | NM_011331 | Ccl12 | 1.6 | 1.0 | 1.3 |  | 1.4 | 2.9 | 3.1 |
| chemokine (C-C motif) ligand 21b (serine) | NM_011124 | Ccl21b | 0.6 | 0.3 | 0.3 |  | 1.1 | 1.3 | 4.1 |
| chemokine (C-C motif) ligand 21c (leucine). | NM_023052 | Ccl21c | 0.6 | 0.3 | 0.3 |  | 1.2 | 1.3 | 3.9 |
| chemokine (C-C motif) ligand 24 | NM_019577 | Ccl24 | 0.6 | 0.7 | 0.6 |  | 6.5 | 10.0 | 6.3 |
| chemokine (C-X-C motif) ligand 1 | NM_008176 | Cxcl1 | 0.8 | 0.7 | 0.6 |  | 2.9 | 17.7 | 12.0 |
| chemokine (C-X-C motif) ligand 9* | NM_008599 | Cxcl9 | 1.5 | 0.5 | 0.5 |  | 17.8 | 8.3 | 2.7 |
| chemokine (C-X-C motif) ligand 13 | NM_018866 | Cxcl13 | 0.9 | 0.4 | 0.3 |  | 3.1 | 20.1 | 7.6 |
| chemokine (C-X-C motif) ligand 14* | NM_019568 | Cxcl14 | - | - | - |  | 0.9 | 4.0 | 9.7 |
| chemokine (C-X3-C motif) ligand 1 | NM_009142 | Cx3cl1 | - | - | - |  | 1.1 | 1.5 | 2.4 |
| chemokine (C motif) ligand 1 | NM_008510 | Xcl1 | 0.7 | 0.2 | 0.2 |  | 2.0 | 2.0 | 1.2 |
| Epstein-Barr virus induced gene 3 | NM_015766 | Ebi3 | 1.4 | 1.5 | 1.8 |  | 1.9 | 4.1 | 3.6 |
| granulin | NM_008175 | Grn | - | - | - |  | 1.4 | 2.2 | 1.6 |
| interferon gamma | NM_008337 | Ifng | - | - | - |  | 1.8 | 2.1 | 1.1 |
| interleukin 11 (Il11) | NM_008350 | Il11 | - | - | - |  | 1.0 | 2.6 | 1.5 |
| interleukin 1 alpha | NM_010554 | Il1a | - | - | - |  | 2.0 | 3.2 | 1.6 |
| oncostatin M (Osm) | XM_137493 | Osm | - | - | - |  | 1.3 | 2.3 | 2.4 |
| tumor necrosis factor | NM_013693 | Tnf | - | - | - |  | 1.6 | 2.6 | 1.9 |
| tumor necrosis factor (ligand) superfamily, member 13 | NM_023517 | Tnfsf13 | 0.7 | 1.1 | 1.3 |  | 1.9 | 4.0 | 4.0 |
| tumor necrosis factor receptor superfamily, member 13b | NM_033622 | Tnfsf13b | - | - | - |  | 1.2 | 3.5 | 6.5 |
| tumor necrosis factor (ligand) superfamily, member 14 | NM_019418 | Tnfsf14 | 0.8 | 1.2 | 1.7 |  | 1.3 | 2.3 | 3.2 |
|  |  |  |  |  |  |  |  |  |  |
| **Cell Adhesion** |  |  |  |  |  |  |  |  |  |
| CD44 antigen* | NM_009851 | Cd44 | - | - | - |  | 2.7 | 5.3 | 4.4 |
| CD6 antigen | NM_009852 | Cd6 | 0.8 | 0.3 | 0.3 |  | 2.8 | 2.1 | 1.7 |
| CD97 antigen | NM_011925 | Cd97 | 0.5 | 0.4 | 0.4 |  | 1.5 | 3.1 | 2.3 |
| cerebral endothelial cell adhesion molecule | NM_207298 | Ceecam1 | 1.3 | 1.6 | 1.2 |  | 1.6 | 2.7 | 3.2 |
| connective tissue growth factor | NM_010217 | Ctgf | 0.6 | 0.4 | 1.1 |  | 0.8 | 1.6 | 3.6 |
| integrin alpha 6 | NM_008397 | Itga6 | - | - | - |  | 0.8 | 2.6 | 2.0 |
| integrin alpha L | NM_008400 | Itgal | - | - | - |  | 2.4 | 3.3 | 2.7 |
| integrin beta 7 | NM_013566 | Itgb7 | 0.6 | 0.4 | 0.4 |  | 2.9 | 4.5 | 4.4 |
| intercellular adhesion molecule | NM_010493 | Icam1 | 0.9 | 0.6 | 0.5 |  | 3.2 | 5.3 | 3.7 |
| intercellular adhesion molecule 2 | NM_010494 | Icam2 | 0.5 | 0.4 | 0.3 |  | 1.8 | 2.7 | 2.0 |
| myelin-associated glycoprotein | NM_010758 | Mag | - | - | - |  | 2.1 | 2.2 | 2.5 |
| neural cell adhesion molecule 1 | NM_010875 | Ncam1 | 0.7 | 1.0 | 1.8 |  | 1.0 | 2.0 | 4.0 |
| platelet/endothelial cell adhesion molecule 1* | NM_008816 | Pecam1 | 0.6 | 0.4 | 0.5 |  | 1.7 | 2.9 | 3.2 |
| selectin, platelet | NM_011347 | Selp | 0.5 | 2.9 | 2.5 |  | 2.0 | 5.1 | 3.4 |
| selectin, platelet (p-selectin) ligand* | NM_009151 | Selpl | - | - | - |  | 3.4 | 7.2 | 8.7 |
| thymus cell antigen 1, theta | NM_009382 | Thy1 | 0.8 | 0.3 | 0.3 |  | 3.2 | 4.3 | 5.1 |
| vascular cell adhesion molecule 1* | NM_011693 | Vcam1 | - | - | - |  | 2.3 | 4.7 | 3.4 |
|  |  |  |  |  |  |  |  |  |  |
| **ECM Components** |  |  |  |  |  |  |  |  |  |
| procollagen, type I, alpha 1 | NM_007742 | Col1a1 | - | - | - |  | 1.3 | 19.3 | 21.6 |
| procollagen, type I, alpha 2 | NM_007743 | Col1a2 | - | - | - |  | 1.1 | 2.4 | 2.6 |
| procollagen, type IV, alpha 1 | NM_009931 | Col4a1 | - | - | - |  | 1.1 | 4.0 | 3.4 |
| procollagen, type IV, alpha 2 | NM_009932 | Col4a2 | - | - | - |  | 1.1 | 3.1 | 3.4 |
| procollagen, type V, alpha 1 | NM_015734 | Col5a1 | 0.7 | 1.1 | 0.9 |  | 1.0 | 5.0 | 4.8 |
| procollagen, type VI, alpha 1* | NM_009933 | Col6a1 | - | - | - |  | 1.3 | 5.5 | 8.6 |
| procollagen, type VI, alpha 2 | NM_146007 | Col6a2 | - | - | - |  | 0.7 | 1.9 | 2.7 |
| procollagen, type XV | NM_009928 | Col15a1 | - | - | - |  | 0.7 | 1.9 | 2.5 |
| elastin | NM_007925 | Eln | - | - | - |  | 1.3 | 1.8 | 2.1 |
| elastin microfibril interfacer 1 | NM_133918 | Emilin1 | 0.9 | 1.2 | 1.3 |  | 1.0 | 2.3 | 1.9 |
| fibrillin 1 | NM_007993 | Fbn1 | - | - | - |  | 1.2 | 3.9 | 7.8 |
| laminin, gamma 1 | NM_010683 | Lamc1 | - | - | - |  | 1.3 | 2.2 | 1.9 |
|  |  |  |  |  |  |  |  |  |  |
| ***Specifically Down-regulated in the Liver*** |  |  |  |  |  |  |  |  |  |
| **Fatty Acid Metabolism** |  |  |  |  |  |  |  |  |  |
| acetyl-Coenzyme A acyltransferase 2 | NM_177470 | Acaa2 | - | - | - |  | 0.8 | 0.5 | 0.4 |
| acetyl-Coenzyme A dehydrogenase, medium chain* | NM_007382 | Acadm | 0.6 | 0.7 | 0.7 |  | 0.7 | 0.5 | 0.3 |
| acyl-Coenzyme A oxidase 1, palmitoyl | NM_015729 | Acox1 | - | - | - |  | 1.0 | 0.6 | 0.4 |
| aldhehyde dehydrogenase family 5, subfamily A1* | NM_172532 | Aldh5a1 | 0.7 | 0.9 | 1.0 |  | 0.5 | 0.4 | 0.3 |
| enoyl coenzyme A hydratase 1, peroxisomal | NM_016772 | Ech1 | - | - | - |  | 0.6 | 0.4 | 0.3 |
| enoyl Coenzyme A hydratase, short chain, 1, mitochondrial | NM_053119 | Echs1 | - | - | - |  | 0.8 | 0.4 | 0.4 |
| fatty acid desaturase 1 | NM_146094 | Fads1 | - | - | - |  | 0.6 | 0.4 | 0.4 |
| fatty acid desaturase 2* | NM_019699 | Fads2 | 0.8 | 1.1 | 1.2 |  | 0.5 | 0.2 | 0.2 |
| lysophospholipase 1 | NM_008866 | Lypla1 | - | - | - |  | 0.9 | 0.5 | 0.4 |
| protein kinase, AMP-activated, alpha 2 catalytic subunit | XM_131633 | Prkaa2 | - | - | - |  | 0.4 | 0.4 | 0.3 |
| solute carrier family 27 (fatty acid transporter), member 2* | NM_011978 | Slc27a2 | - | - | - |  | 0.5 | 0.3 | 0.2 |
| solute carrier family 27 (fatty acid transporter), member 5 | NM_009512 | Slc27a5 | - | - | - |  | 0.7 | 0.4 | 0.4 |
|  |  |  |  |  |  |  |  |  |  |
| **Amino acid metabolism** |  |  |  |  |  |  |  |  |  |
| acyl-Coenzyme A dehydrogenase family, member 8* | NM_025862 | Acad8 | - | - | - |  | 0.9 | 0.5 | 0.4 |
| acetyl-Coenzyme A dehydrogenase, medium chain* | NM_007382 | Acadm | 0.6 | 0.7 | 0.7 |  | 0.7 | 0.5 | 0.3 |
| aminoacylase 1 | NM_025371 | Acy1 | - | - | - |  | 0.5 | 0.3 | 0.3 |
| aldehyde dehydrogenase 4 family, member A1 | NM_175438 | Aldh4a1 | 0.8 | 0.9 | 1.0 |  | 0.5 | 0.4 | 0.3 |
| aldhehyde dehydrogenase family 5, subfamily A1* | NM_172532 | Aldh5a1 | 0.7 | 0.9 | 1.0 |  | 0.5 | 0.4 | 0.3 |
| argininosuccinate lyase* | NM_133768 | Asl | - | - | - |  | 0.7 | 0.4 | 0.3 |
| argininosuccinate synthetase 1 | NM_007494 | Ass1 | - | - | - |  | 0.9 | 0.3 | 0.3 |
| branched chain ketoacid dehydrogenase E1, alpha polypeptide | NM_007533 | Bckdha | - | - | - |  | 0.5 | 0.4 | 0.3 |
| cystathionine beta-synthase (Cbs), transcript variant 1* | NM_144855 | Cbs | - | - | - |  | 0.5 | 0.4 | 0.3 |
| catechol-O-methyltransferase. | XM_147265 | Comt | - | - | - |  | 1.0 | 0.7 | 0.5 |
| dopa decarboxylase | NM_016672 | Ddc | - | - | - |  | 1.1 | 0.6 | 0.3 |
| dihydrofolate reductase | NM_010049 | Dhfr | - | - | - |  | 0.6 | 0.4 | 0.3 |
| fumarylacetoacetate hydrolase* | NM_010176 | Fah | - | - | - |  | 0.6 | 0.5 | 0.4 |
| glycine decarboxylase | NM_138595 | Gldc | - | - | - |  | 0.8 | 0.6 | 0.3 |
| glutaminase 2 (liver, mitochondrial) | XM_125928 | Gls2 | - | - | - |  | 0.3 | 0.2 | 0.1 |
| glutamate oxaloacetate transaminase 2, mitochondrial* | NM_010325 | Got2 | - | - | - |  | 0.6 | 0.5 | 0.4 |
| glutathione transferase zeta 1 (maleylacetoacetate isomerase) | NM_010363 | Gstz1 | - | - | - |  | 0.8 | 0.5 | 0.4 |
| 3-hydroxyisobutyrate dehydrogenase | NM_145567 | Hibadh | - | - | - |  | 0.7 | 0.4 | 0.3 |
| N-acetylglutamate synthase* | NM_178053 | Nags | 1.0 | 1.4 | 1.0 |  | 0.6 | 0.6 | 0.5 |
| prenylcysteine oxidase 1 | NM_025823 | Pcyox1 | - | - | - |  | 0.6 | 0.5 | 0.4 |
| proline dehydrogenase (oxidase) 2* | NM_011172 | Prodh | - | - | - |  | 0.7 | 0.3 | 0.2 |
| serine hydroxymethyl transferase 1 (soluble)* | NM_009171 | Shmt1 | - | - | - |  | 0.5 | 0.2 | 0.2 |
| serine hydroxymethyl transferase 2 (mitochondrial) | NM_028230 | Shmt2 | 1.1 | 2.2 | 1.6 |  | 0.7 | 0.5 | 0.4 |
| serine racemase* | NM_013761 | Srr | - | - | - |  | 0.6 | 0.4 | 0.3 |
|  |  |  |  |  |  |  |  |  |  |
| ***Enhanced in Spleen*** |  |  |  |  |  |  |  |  |  |
| **Porphyrin and haem biosynth** |  |  |  |  |  |  |  |  |  |
| ferrochelatase* | NM_007998 | Fech | 2.1 | 2.1 | 2.5 |  | 0.7 | 0.6 | 0.5 |
| hydroxymethylbilane synthase | NM_013551 | Hmbs | 2.1 | 2.4 | 2.0 |  | 0.6 | 0.6 | 0.4 |
| protoporphyrinogen oxidase* | NM_008911 | Ppox | 2.2 | 2.6 | 2.4 |  | - | - | - |
| uroporphyrinogen III synthase* | NM_009479 | Uros | 2.1 | 2.5 | 1.7 |  | 1.0 | 0.8 | 0.6 |
|  |  |  |  |  |  |  |  |  |  |
|  |  |  |  |  |  |  |  |  |  |
| ***Specifically Down-regulated in the spleen*** |  |  |  |  |  |  |  |  |  |
| **B-cell/T-cell Activation** |  |  |  |  |  |  |  |  |  |
| B-cell scaffold protein with ankyrin repeats 1 | XM_143587 | Bank1 | 0.7 | 0.4 | 0.4 |  | - | - | - |
| B-cell leukemia/lymphoma 6 | NM_009744 | Bcl6 | 0.7 | 0.5 | 0.4 |  | 0.8 | 1.9 | 1.6 |
| Burkitt lymphoma receptor 1* | NM_007551 | Blr1 | 0.9 | 0.5 | 0.4 |  | - | - | - |
| CD2 antigen | NM_013486 | Cd2 | 0.7 | 0.3 | 0.3 |  | 1.5 | 1.6 | 1.3 |
| CD28 antigen* | NM_007642 | Cd28 | 0.9 | 0.4 | 0.4 |  | 1.5 | 1.4 | 1.2 |
| c-src tyrosine kinase | NM_007783 | Csk | 1.0 | 0.6 | 0.5 |  | 0.8 | 1.3 | 0.9 |
| chemokine (C-X-C motif) ligand 12, transcript variant 2.* | NM_021704 | Cxcl12 | 0.5 | 0.4 | 0.5 |  | 0.7 | 0.7 | 0.8 |
| early growth response 1 | NM_007913 | Egr1 | 0.8 | 0.5 | 0.4 |  | 0.8 | 1.9 | 2.1 |
| forkhead box P1* | NM_053202 | Foxp1 | 0.6 | 0.4 | 0.4 |  | 0.8 | 0.9 | 0.9 |
| histone deacetylase 7A | NM_019572 | Hdac7a | 0.7 | 0.5 | 0.5 |  | 1.0 | 1.5 | 1.9 |
| icos ligand | NM_015790 | Icosl | 0.7 | 0.4 | 0.6 |  | 1.1 | 1.3 | 1.6 |
| interleukin 18* | NM_008360 | Il18 | 0.9 | 0.6 | 0.6 |  | 1.0 | 0.9 | 0.6 |
| interleukin 27 receptor, alpha | NM_016671 | Il27ra | 0.8 | 0.4 | 0.3 |  | 1.3 | 1.6 | 1.2 |
| interleukin 2 receptor, gamma chain | NM_013563 | Il2rg | 0.6 | 0.4 | 0.3 |  | 1.5 | 1.2 | 1.0 |
| interleukin 7 | NM_008371 | Il7 | 0.4 | 0.3 | 0.3 |  | 1.6 | 1.6 | 1.6 |
| mucosa associated lymphoid tissue lymphoma translocation gene 1 | NM_172833 | Malt1 | 0.7 | 0.5 | 0.4 |  | - | - | - |
| membrane-spanning 4-domains, subfamily A, member 1 | NM_007641 | Ms4a1 | 0.7 | 0.4 | 0.4 |  | 1.0 | 1.0 | 1.0 |
| NK2 transcription factor related, locus 3 (Drosophila) | NM_008699 | Nkx2-3 | 0.6 | 0.5 | 0.5 |  | - | - | - |
| sialophorin | AK041480 | Spn | 0.6 | 0.5 | 0.4 |  | 0.9 | 1.0 | 1.0 |
| SWAP complex protein, 70 kDa* | NM_009302 | Swap70 | 0.8 | 0.5 | 0.5 |  | 1.0 | 1.5 | 1.4 |
|  |  |  |  |  |  |  |  |  |  |
| **Cytokine binding** |  |  |  |  |  |  |  |  |  |
| Burkitt lymphoma receptor 1* | NM_007551 | Blr1 | 0.9 | 0.5 | 0.4 |  | - | - | - |
| chemokine binding protein 2 | NM_021609 | Ccbp2 | 0.7 | 0.5 | 0.4 |  | 1.5 | 2.0 | 1.8 |
| chemokine (C-C motif) receptor 6* | NM_009835 | Ccr6 | 1.0 | 0.5 | 0.5 |  | - | - | - |
| chemokine (C-C motif) receptor 7 | NM_007719 | Ccr7 | 0.7 | 0.2 | 0.3 |  | 1.6 | 1.3 | 1.4 |
| interleukin 2 receptor, gamma chain | NM_013563 | Il2rg | 0.6 | 0.4 | 0.3 |  | 1.5 | 1.2 | 1.0 |
| interleukin 7 receptor* | NM_008372 | Il7r | 0.5 | 0.3 | 0.4 |  | 1.6 | 1.6 | 1.6 |
| interleukin 18 receptor 1 | NM_008365 | Il18r1 | 0.7 | 0.4 | 0.5 |  | 1.0 | 1.3 | 1.2 |
| interleukin 21 receptor | NM_021887 | Il21r | 0.7 | 0.5 | 0.4 |  | 1.4 | 1.6 | 1.5 |
| interleukin 27 receptor, alpha | NM_016671 | Il27ra | 0.8 | 0.4 | 0.3 |  | 1.3 | 1.6 | 1.2 |
| latent transforming growth factor beta binding protein 4 | NM_175641 | Ltbp4 | 0.5 | 0.3 | 0.4 |  | 0.8 | 0.8 | 1.0 |
|  |  |  |  |  |  |  |  |  |  |
